# Synthesis, Characterization, and Biological Activity Studies of Copper(II) Mixed Compound with Histamine and Nalidixic Acid

**DOI:** 10.1155/2009/603651

**Published:** 2009-06-18

**Authors:** Egla Yareth Bivián-Castro, Mercedes G. López, Mario Pedraza-Reyes, Sylvain Bernès, Guillermo Mendoza-Díaz

**Affiliations:** ^1^Departamento de Química, División de Ciencias Naturales y Exactas, Universidad de Guanajuato, Noria Alta s/n, 36050 Guanajuato, GTO, Mexico; ^2^Centro Universitario de los Lagos, Universidad de Guadalajara, Avenida Enrique Díaz de León 1144 Col. Paseos de la Montaña, Lagos de Moreno, 47460 Jalisco, JAL, Mexico; ^3^Departamento de Biotecnología y Bioquímica, Cinvestav-IPN Unidad Irapuato, Km.9.6 Libramiento Norte, Carr. Irapuato-León, 36500 Irapuato, GTO, Mexico; ^4^Departamento de Biología, División de Ciencias Naturales y Exactas, Universidad de Guanajuato, Noria Alta s/n, 36050 Guanajuato, GTO, Mexico; ^5^DEP Facultad de Ciencias Químicas, UANL, Guerrero y Progreso s/n, Col. Treviño, 64570 Monterrey, NL, Mexico

## Abstract

A mixed copper complex with deprotonated nalidixic acid (nal) and histamine (hsm) was synthesized and characterized by FTIR, UV-Vis, elemental analysis, and conductivity. The crystal structure of [Cu(hsm)(nal)H_2_O]Cl·3H_2_O (**chn**) showed a pentacoordinated cooper(II) in a square pyramidal geometry surrounded by two N atoms from hsm, two O atoms from the quinolone, and one apical water oxygen. Alteration of bacterial DNA structure and/or associated functions in vivo by [Cu(hsm)(nal)H_2_O]Cl·3H_2_O was demonstrated by the induction of a *recA-lacZ* fusion integrated at the *amyE* locus of a recombinant *Bacillus subtilis* strain. Results from circular dichroism and denaturation of calf thymus DNA (CT-DNA) suggested that increased amounts of copper complex were able to stabilize the double helix of DNA in vitro mainly by formation of hydrogen bonds between **chn** and the sugars of DNA minor groove. In vivo and in vitro biological activities of the **chn** complex were compared with the chemical nuclease [Cu(phen)(nal)H_2_O]NO_3_ · 3H_2_O (**cpn**) where phen is phenanthroline.

## 1. Introduction

**Figure 1:**
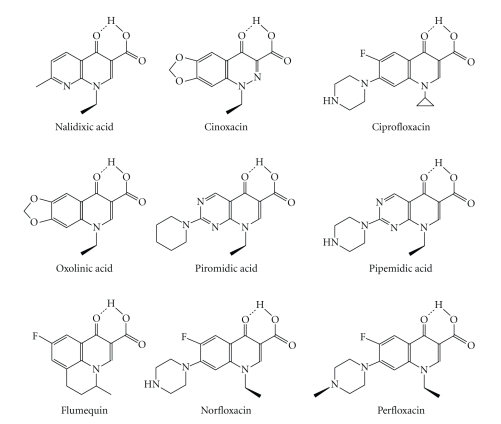
Some quinolone and fluoroquinolone drugs chemical structures.

Since Lescher and col. prepared the nalidixic acid in 1962 [1], the synthesis of new quinolones continues (Figure 1). First generation antibiotics of quinolone family are effective against gram-negative bacteria usually found in light urinary diseases [2]; examples of these drugs are Nalidixic acid, Oxolinic acid and Cinoxacin. The fluoroquinolones, second generation, like ciprofloxacin are also effective against gram-positive bacteria; they can be used in urinary, skin, or respiratory infections [3]. Levofloxacin and sparfloxacin are examples of third generation quinolones, also known as fluoroquinolones, but they are more effective against gram-positives organism. They are used in treatment of pneumonia, bronchitis, sinusitis, and gonorrhea infections.

Fourth generation quinolones include the trovofloxacin with an effectiveness against anaerobic organism [4]. Quinolones inhibit the bacterial DNA synthesis [2], and there are several hypotheses to explain their mechanism of action. One of these hypothesis suggests the inhibition of subunit A of DNA-gyrase in presence of ATP [5–7]. Another hypothesis supports the existence of a cooperative bond between the quinolone–DNA–DNA-gyrase [8–11]. Also, it is suggested that quinolone may form coordination complexes with a transition metal ion present in the cytoplasm or with a metal biocomplex; this metal ion could be copper(II) [12]. Copper in biological systems is usually found surrounded by imidazole moieties from histidine residues [13, 14]. Copper concentration increases when a disease like arthritis or a neoplasia is present; therefore under the stress caused by the disease, copper may be available for coordination with some endogenic or exogenic compounds like peptides and drugs, respectively. With this in mind, the interactions of different quinolones with copper and other metal ions have been investigated for years [15]. The hsm-copper(II) system may be used to mimic the environment of this metal ion in some biological systems. The histamine molecule is a good ligand, which contains not only N atoms (from imidazole ring and aliphatic ammine) but also hydrogen atoms that are good for hydrogen bond formation and therefore are useful in molecular recognition. Because of these characteristics some histamine complexes with copper have been reported recently [16, 17]. In the present work we reported the synthesis, characterization, and biological activity results of a copper mixed complex (see Figure 1), with hsm and nalidixic acid. This study contributes to get better knowledge how a metal ion is required to carry the quinolones to their site of action and also to clarify how metal surroundings affect the biological action of quinolones drugs.

## 2. Experimental

### 2.1. Materials and Physical Measurements

The histamine dihydrochloride, nalidixic acid (Sigma-Aldrich products), and CuCl_2_ · 2H_2_O (J. T. Baker products) were used as provided. Far-IR spectra were taken on a Bruker IFS-55 spectrophotometer as polyethylene pellets. Medium-IR spectra were carried out on Perkin-Elmer spectrophotometer model. FT 1600 as KBr pellets. Electronic spectra were recorded on an Agilent 8453 UV-Vis spectrophotometer using water as solvent. The molar conductivity measurements were made with a Conductronic PC18 conductivity bridge with a nickel-platinezed electrodes cell using water solutions prepared immediately before used. Elemental analyses were performed by Desert Analysis, Organic Microanalysis, Tuczon AZ, USA. 

### 2.2. Synthesis of Mixed Complexes

[Cu(hsm)(nal)H_2_O]Cl · 3H_2_O: Nalidixic acid (232.2 mg, 1 mmol) was dissolved with NaOH (40 mg, 1 mmol) in 70 mL of water. This solution was added into a previously prepared equimolar mixture of histamine dihydrochloride (184.07 mg, 1 mmol) and CuCl_2_ · 2H_2_O (170.48 mg, 1 mmol) dissolved in a total volume of 15 mL of water. The final solution was brought to pH 7.5 and left for slow crystallization in a refrigerator (4°C). Purple crystals suitable for X-ray analysis were isolated. *Anal.* Calc. for C_17_H_31_CuN_5_O_8_Cl: C, 38.35; H, 5.87; N, 13.15. Found: C, 39.82; H, 5.50; N, 13.65%. IR (KBr, cm^−1^): 1634(s), 1607(m), 1522(m), 1501(m), 539 (s), 503 (m). UV-Vis (H_2_O, nm): *λ*_max _ 212 (*ε*_212_ = 25674 L mol^−1^ cm^−1^), 257 (*ε*_257_ = 25596 L mol^−1^ cm^−1^), 323 (*ε*_323_ = 11138.15 L mol^−1^ cm^−1^), 625 (*ε*_625_ = 63.47 L mol^−1^ cm^−1^). Conductivity: 105 Ω^−1^ cm^2^ mol^−1^ in water 10^−3^ M.

[Cu(phen)(nal)H_2_O]NO_3_ · 3H_2_O was obtained as previously reference indicates [18].

### 2.3. Crystallographic Studies

A crystal specimen of 0.35 × 0.20 × 0.15 mm^3^ was selected and mounted on a Siemens P4/PC diffractometer using highly oriented graphite monochromatized Mo-*K*_*α*_ radiation, *λ* = 0.71073Å, *T* = 293 K; none absorption correction was made. The structure was solved by the direct methods (SIR92) and difference Fourier maps and refined by the full-matrix least squares. C_17_H_28_Cu_1_Cl_1_N_5_O_7_, *M* = 513.43, Blue, regular prism, triclinic crystals, space group, $\;\text{P}\overset{̅}{1}$, a = 10.4745(17)Å, b = 10.5001(16)Å, c = 11.1374(19)Å, *α* = 80.408(10)°, *β* = 85.184(10)°, *γ* = 72.541(10)°, *V* = 1151.4(3)Å^3^, *Z* = 2, *ρ*_calc_ = 1.481 Mg/m^3^, *μ* = 1.111mm^−1^, 4796 reflections collected (1.86 < *θ* < 25.00°, *R*_int_ = 0.0226), Final *R* indices [*I* > 2*σ* (*I*)], *R*_1_ = 0.0455, _*w*_ *R*_2_ = 0.1053, *R* indices (all data), *R*_1_ = 0.0655, _*w*_ *R*_2_ = 0.1161. Selected bonds lengths and bond angles are presented in Table 1. All data are available at Cambridge Crystallographic Data Centre; see supplementary material.

### 2.4. Biological Test

#### 2.4.1. Organism and Growth Conditions

*B. subtilis* strain YB-3001 carrying pCCR202 (a *rec*A-*lac*Z fusion integrated in the *amy*E locus) was kindly provided by Dr. Ronald E. Yasbin (University of Nevada, Las Vegas). To propagate this strain, Luria Bertani (LB) medium was used. Chloramphenicol was added to the culture medium at a final concentration of 3 *μ*g/mL.

#### 2.4.2. Induction Experiments

*B. subtilis* YB-3001 was grown to an optical density of 0.3 at 600 nm, and at this point, the culture was split into 6 subcultures. One of the cultures was used as control, and the other five were supplemented with CuCl_2_, nalidixic acid, [Cu(hsm)]^2+^, hsm, and [Cu(hsm)(nal)H_2_O]Cl · 3H_2_O complex to a final concentration of 300 *μ*M. Samples of the culture were collected before and after 5 hours of addition of the compounds and they were processed for *β*-galactosidase activity as previously described [19, 20]. The *β*-galactosidase activity is reported in Miller units [21].

#### 2.4.3. Nuclease Activity of Metal Complexes Against a 196 **b** **p** DNA Fragment

Experiments to characterize the nuclease activity of the metal complex were performed using as a substrate a 196 **bp** PCR fragment encompassing part of the open reading frame and the promoter regions of the *ytk*D gene from *B*. *subtilis* [22]. Amplification of this DNA fragment was carried out using 0.1 *μ*g of chromosomal DNA from *B. subtilis* 168 and the oligonucleotide primers 5′-GGGATAAACATGTACGAG-3′ (forward) and 5′-CTTCTG CGCACTCCAT CGGCTCTAC-3′ (reverse). Amplification was performed with Vent DNA polymerase (New England Biolabs, Beverly, MA). The 196 bp DNA obtained was purified from low melting point agarose gels as previously described [23].

A typical reaction mixture contained in a final volume of 25 *μ*L: DNA 375 ng, [Cu(hsm)(nal)H_2_O]Cl · 3H_2_O or [Cu(phen)(nal)H_2_O]NO_3_ · 3H_2_O complexes, 100 *μ*M, and Mercaptopropionic acid (MPA), 7 mM. The reaction mixture was incubated for 50 minutes at 37°C, diluted with loading buffer, which contained 50 mM Tris-acetate buffer, pH 7, glycerol 50% (v/v), and Bromophenol blue as a tracking dye, and loaded on a 2% agarose gel containing 50 mM Tris-acetate buffer, pH 7, and 1 mg/mL ethidium bromide. The gel was run in the same buffer at 90 V. The DNA on the gel was observed and photographed using an Eagle Eye gel documentation system. 

#### 2.4.4. Biophysical Studies

Sodium cacodylate hydrate and Calf Thymus DNA (CT-DNA) were purchased from Sigma-Aldrich Chemical Company. CT-DNA was dialyzed at 4°C for purification against a buffer of 5 mM sodium cacodylate, 0.6 M NaCl, pH = 7.1. The ratio A_280_/Abs_260_ = 2 was used as an indication of a DNA free of proteins. The concentration of the CT-DNA stock solution was 6.22x10^−3^ M, determined at 260 nm and applying the molar extinction coefficient of 6600 M^−1^ cm^−1^ given in literature [24].

#### 2.4.5. CD Spectra

CD spectra were recorded at increasing [Cu(hsm)(nal)H_2_O]Cl · 3H_2_O /CT-DNA ratio (*r* = 0.0, 0.048, 0.14, 0.24, 0.38, 0.48). The DNA concentration was kept equal to 2.07 × 10^−4^ M in all experiments. After 1 hour of incubation at 37°C, the spectrum of each sample was recorded on a Jasco—715 spectropolarimeter at room temperature under constant nitrogen flush. The wavelength range between 200 and 300 nm was monitored. 

All CD spectra were carried out in 5 mM sodium cacodylate, 0.6 M NaCl, pH = 7.1 buffer, using a quartz cell. Similar experiments were made with [Cu(phen)(nal)H_2_O]NO3 · 3H_2_O complex. The sodium cacodylate buffer was employed as background.

#### 2.4.6. Melting Temperature Studies

Thermal denaturation experiments were performed in the same buffer as the CD spectra, using quartz cuvettes with an Agilent 8453 UV-visible spectrophotometer equipped with a Peltier system. The chn/CT-DNA molar ratio was *r* = 0.0, 0.096, 0.28, 0.48, 0.77, and 0.96. After 1 hour of incubation at 37°C, samples were continuously heated at 1°C min^−1^. As the temperature was increasing, the absorbance changes at 260 nm were recorded. The investigated interval of temperature ranged from 37°C to 90°C. DNA concentration was equal to 1.03 × 10^−4^ M. Similar experiments were made with the **cpn** complex. 

## 3. Results and Discussions

### 3.1. Structural and Spectroscopic Properties of Mixed Complexes

**Figure 2:**
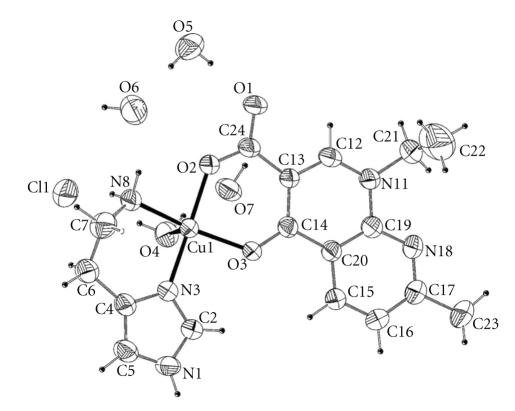
ORTEP plot showing the molecular structure of [Cu(hsm)(nal)H_2_O]Cl · 3H_2_O complex. Ellipsoids are at 50% probability.

The molecular structure of [Cu(hsm)(nal)H_2_O]Cl · 3H_2_O complex of Figure 2  shows a distorted square pyramidal geometry around the copper ion as it is suggested by the distortions parameter that * τ*. Tau-descriptor (*τ*) for five coordination complexes expressed as the difference between the angles of the bonds O(2)–Cu(1)–N(3) and O(3)–Cu(1)–N(8) divided by 60 gives a value of 0.13, being the ideal values of 1 for a trigonal bipyramid and 0 for a square pyramid [25]. 

**Figure 3:**
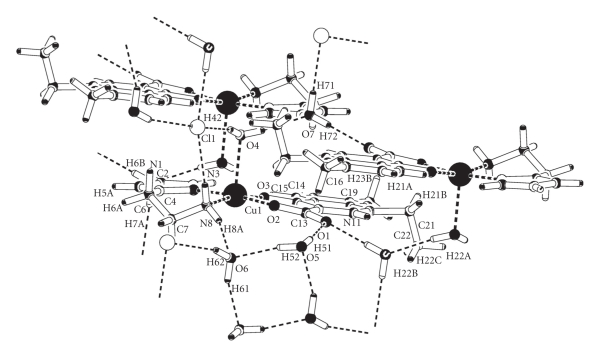
PLUTON plot showing the stacking between nalidixate rings and hydrogen bondings in the crystal of [Cu(hsm)(nal)H_2_O]Cl · 3H_2_O complex.

The base of the pyramid is formed by two oxygen atoms of the nalidixate ligand (one oxygen of the carboxylate group and other for the keto group, Cu(II)–O(2) = 1.934(3), Cu(II)–O(3)=1.961(2) Å resp.) and two N atoms of the histamine ligand (one aliphatic nitrogen and other imidazole nitrogen, Cu–N(8) = 1.996(3) Å, Cu–N(3) = 1.966(3) Å resp.). The C(24)–O(1) bond length is longer than the C(24)–O(2) as expected for carboxylic metal coordination. Copper and O(3), O(2), and N(3) are almost coplanar, and they define the mean square plane of the pyramid base (0.6022(4)*X* + 0.5019(3)*Y* + 0.6208(3) = 16.1191(18)). The major deviations of those atoms are −0.077 Å whereas the N(8), that is, also forming part of the pyramid base is about 0.244(3) Å above the plane. At apical position a molecule of water is coordinated (Cu–O(4) = 2.344(3) Å). The angle formed between the mean plane of the pyramid base and the apical ligand is 87.73(12)°. The amino group of histamine is coordinated in trans position to the keto group analogous to copper complex reported previously [26]. In Figure 3, it is possible to observe that the packing pattern is largely dictated by *π* − *π* stacking forces interaction between the aromatic rings of the nalidixate anion with a distance of 3.771 Å as well as hydrogen bonding interactions since crystal structure includes an uncoordinated chloride anion and three water molecules that provide crystalline stability throught a hydrogen bond network. 

The IR spectra of [Cu(hsm)(nal)**)**H_2_O]Cl · 3H_2_O compound are quite complex due to the presence of numerous functional groups in the molecules; selected signals are listed in Section 2. The mid-infrared spectrum showed bands corresponding to both ligands. A broad band around 3500 cm^−1^ can be assigned to the O–H stretching vibrations of lattice water molecules. Also between 3261 and 2661 cm^−1^ some medium bands appeared corresponding to amine and amide vibrations forms. A broad band between 1634 and 1501 cm^−1^ which is splitted and includes the vibrations *v*_*α*_ (COO) and *v*_*s*_ (CO) is present, showing the interaction of these groups (the 4-oxo and to the 3-carboxylate groups) with the metallic ion. Similar results are observed for far infrared spectrum. At 538 and 503 cm^−1^ two bands assigned to Cu–NH_2_ combination modes appeared, as it had reported previously [26]. The UV-visible spectra showed a broad band centered around 625 nm, that is, observed due to the d-d transitions for Cu(II) corresponding to a typical square pyramidal copper(II) complex with two nitrogen atoms coordinated. In the UV region three intense bands appeared that correspond to the *π* − *π* transitions of the ligands as showed in similar hsm complex previously reported [26]. It is remarkable the similarity of chemical structure of [Cu(hsm)(nal)H_2_O]Cl · 3H_2_O compound with [Cu(phen)(nal)H_2_O]NO_3_ · 3H_2_O complex [18]. While **cpn** complex has a desirable planarity due to phenanthroline ligand, on the other hand **chn** complex has the possibility to form hydrogen bonding due to histamine ligand. 

### 3.2. Biological Activity Studies of [Cu(hsm)(nalH_2_OCl · 3H_2_O Comparing with [Cu(phen)(nalH_2_ONO_3_ · 3H_2_O

**Figure 4:**
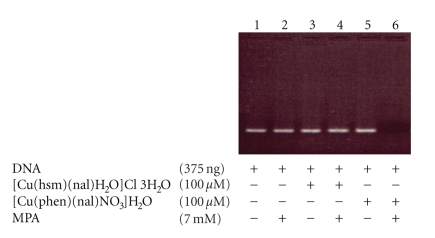
Activity of [Cu(hsm)(nal)H_2_O]Cl · 3H_2_O for cleavage of plasmid DNA. 375 ng of 196 bp DNA were incubated with (lanes 3, 4) or without (lanes 1, 2) [Cu(hsm)(nal)H_2_O]Cl · 3H_2_O (100 *μ*M) or with (lanes 5, 6) [Cu(phen)(nal)H_2_O]NO_3_ · 3H_2_O (100 *μ*M) in presence (lanes 2, 4, 6) or absence (lanes 1, 3, 5) of mercapto propionic acid, (MPA) (7 Mm), for 50 minutes at 37°C.

The ability of the [Cu(phen)(nal)H_2_O]NO_3_ · 3H_2_O metal complex to interact with DNA in vivo and its capacity to promote the complete degradation of plasmid and chromosomal DNA under reductive conditions has been previously reported [27]. Therefore we investigated whether the [Cu(hsm)(nal)H_2_O]Cl · 3H_2_O complex has similar biological activity. For this purpose, we investigated here whether **chn** complex behaves as a chemical nuclease by inducing the degradation of double stranded DNA under both, reductive, and nonreductive conditions. As a positive control we used **cpn** complex at the same concentration. The results shown on Figure 4  revealed that the **chn** complex does not posses chemical nuclease activity even in the presence of Mercaptopropionic acid (MPA) (Figure 4, lanes 3 and 4). On the other hand, as previously reported the **cpn** complex behaved as a thiol-dependent chemical nuclease with a complete degradation of DNA at this concentrations (Figure 4, Lane 6) [27]. 

**Figure 5:**
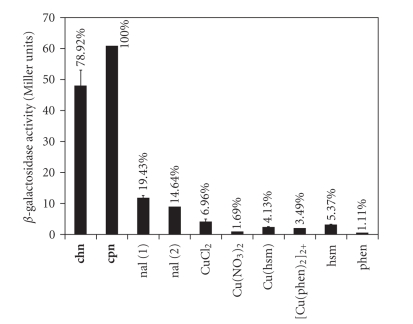
Maximum levels of *rec*A-directed *β*-galactosidase induction obtained as response of the addition of the [Cu(hsm)(nal)H_2_O]Cl · 3H_2_O (chn), [Cu(phen)(nal)H_2_O]NO_3_ · 3H_2_O, (cpn) complexes, or some of their molecular components on cultures of *B. subtilis* YB3001. Percentage indicated as comparison to the highest activity encountered (cpn = 100%).

To investigate the interaction of **chn** with DNA we took advantage of the bacterial SOB response of the gram-positive microorganism *Bacillus subtilis*. This response, similar in several aspects to the SOS response of *E. coli*, is triggered by physicochemical agents which promote alterations in the structure of DNA and when *B. subtilis* is grown under conditions which induce genetic competence [27–29]. Thus the strain *B. subtilis* YB3001, containing a single copy of the *rec*A*-lac*Z fusion integrated at the *amyE *locus, was treated with [Cu(hsm)(nal)H_2_O]Cl · 3H_2_O complex and with each of its components, (CuCl_2_, hsm, nal, and [Cu(hsm)]^2+^). As shown in Figure 5  the levels of *β* -galactosidase induced by the [Cu(hsm)(nal)H_2_O]Cl · 3H_2_O complex were 20% lower than those induced by the analogous [Cu(phen)(nal)H_2_O]NO_3_ · 3H_2_O complex [27]. However it is noticeably that none of the components of the [Cu(hsm)(nal)H_2_O]Cl · 3H_2_O complex were able to induce the *β*-galactosidase activity from the *rec*A-*lac*Z fusion to levels comparable to those observed for the full complex, neither its activity can be interpreted as an additive effect of the independent components. 

Therefore, we can conclude that the complex acts as a whole, being able to cross the cell membranes and interact with the DNA directly. 

**Figure 6:**
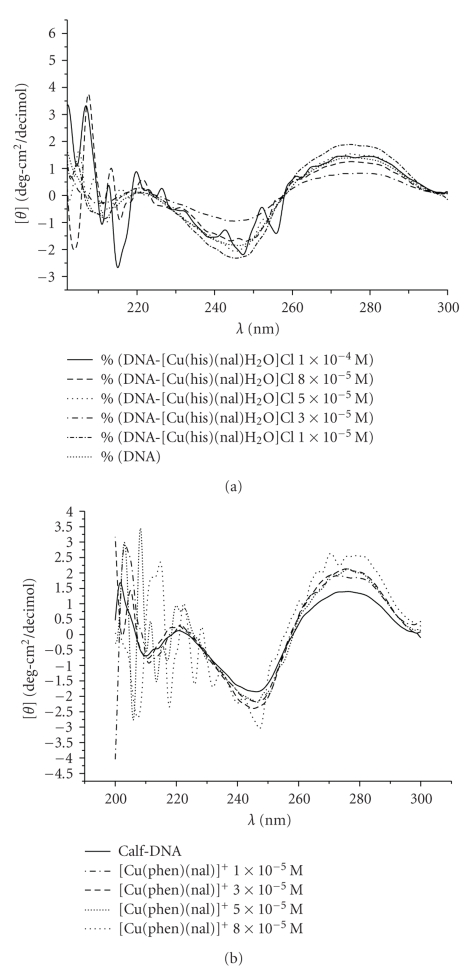
CD titration of CT-DNA with increasing amounts of the [Cu(hsm)(nal)H_2_O]Cl · 3H_2_O (a) and [Cu(phen)(nal)H_2_O]NO_3_ · 3H_2_O (b) complexes at the following molar ratios: *r* = 0.0, 0.048, 0.14, 0.24, 0.38, and 0.48. (Complex/DNA). [DNA] = 2.07*x*10^−4^ M for all experiments.

To investigate the way of [Cu(hsm)(nal)H_2_O]Cl · 3H_2_O DNA interaction, the in vitro interaction of **chn** and **cpn **with CT-DNA was analyzed by melting temperature and circular dichroism (CD). In Figure 6  it is shown the CD spectra of CT-DNA following additions of increasing amounts of [Cu(hsm)(nal)H_2_O]Cl · 3H_2_O. 

**Figure 7:**
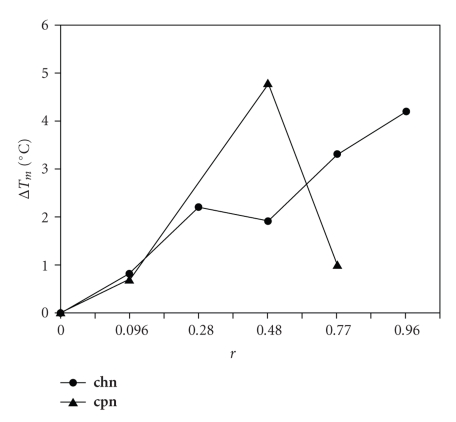
Calf thymus DNA ΔTm values at increasing amounts of [Cu(hsm)(nal)H_2_O]Cl · 3H_2_O and [Cu(phen)(nal)H_2_O]NO_3_ · 3H_2_O complexes.

Results reveal that this complex induces small CD changes. Similar behavior was found for complex [Cu(phen)(nal)H_2_O]NO_3_ · 3H_2_O. It is important to mention that both complexes are CD inactive. The observed changes in the CD spectra suggest that both complexes interact with DNA through the sugar-phosphate bone, in the minor groove. This result is in agreement with the increment in the melting point observed when the complex concentration increases, (see Figure 7) and suggests that the main interaction between the complexes and DNA chain may be trough hydrogen bond interactions. The results showed that at low concentration both complexes increase the T_m_ of CT-DNA (Table 2). However at higher concentrations a different behavior between both complexes was observed. 

This difference between both complexes should be related with the type of coordinated ammine. Being histamine able to form stronger hydrogen bonds, it is reasonable to speculate that when complex concentration increases, more hydrogen bonds will be formed and therefore more stable the double helix will be. On the other hand, when phenantroline is the diammine at low concentration, it is possible that the main interaction between the complex and DNA is dominated by hydrogen bonds with the antibiotic, but when concentration increases, it is possible to have two types of interactions between the complex and DNA, one via hydrogen bonds as stated before and a second one weaker due to the intercalation of the phenanthroline rings, which will be reflected in a destabilization of the double helix. In conclusion, from this study is possible to say that quinolones behave very similar as ligands; also it is observed that their coordination mode is relatively independent of the type of coordinated diammine. However the activity as chemical nuclease is strongly dependent of the diammine. Our work suggests that complexes of the quinolone family drugs with copper coordinated to other ammines may be active, even if the nuclease activity is not present as in those complexes with phenantroline. It seems that DNA-complex interaction is dominated by hydrogen-bond type, and therefore the design of new potential copper-based drugs should consider this.

## Figures and Tables

**Scheme 1:**
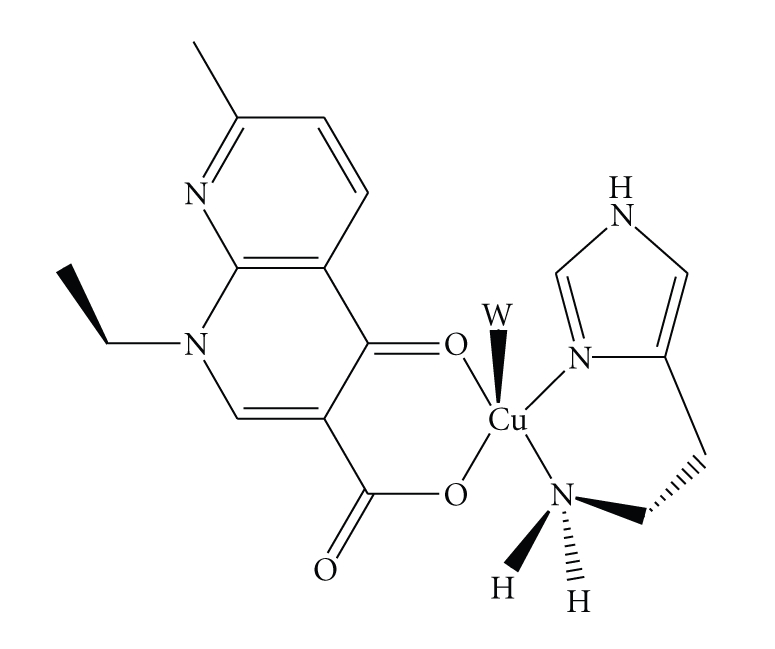
Synthesis, characterization and crystal structure of a copper(II) mixed complex with histamine and nalidixic acid, a compound of the quinolone family, [Cu(hsm)(nal)H_2_O]Cl∙3H_2_O is reported. Biological studies performed with this compound revealed that it interacts with DNA *in vivo* inducing the SOB response on *Bacillus subtilis*, however nuclease activity is not observed in vitro experiments

**Table 1:** Selected bond lengths (Å) and bond angles (∘) for [Cu(hsm)(nal)H_2_O]Cl · 3H_2_O.

Bond lengths (Å)		Bond angles (∘)	
Cu(1)–O(2)	1.934(3)	O(2)–Cu(1)–O(3)	91.22(12)
Cu(1)–O(3)	1.961(2)	O(2)–Cu(1)–N(3)	173.72(14)
Cu(1)–N(3)	1.966(3)	O(3)–Cu(1)–N(3)	90.39(12)
Cu(1)–N(8)	1.996(3)	O(2)–Cu(1)–N(8)	84.84(14)
Cu(1)–O(4)	2.344(3)	O(3)–Cu(1)–N(8)	165.77(11)
N(3)–C(4)	1.385(4)	N(3)–Cu(1)–N(8)	92.14(13)
N(8)–C(7)	1.479(5)	O(2)–Cu(1)–O(4)	93.47(13)
O(1)–C(24)	1.241(4)	O(3)–Cu(1)–O(4)	95.56(10)
O(2)–C(24)	1.264(4)	N(3)–Cu(1)–O(4)	92.41(12)
O(3)–C(14)	1.271(4)	N(8)–Cu(1)–O(4)	98.32(12)
C(4)–C(6)	1.490(6)		
C(6)–C(7)	1.510(6)		
C(13)–C(14)	1.419(5)		
C(13)–C(24)	1.484(5)		

**Table 2:** Expermental melting temperatures (Tm) and difference with the CT-DNA Tm (ΔTm) values as determined from the thermal denaturation profiles of CT-DNA and its interactions with the [Cu(hsm)(nal)H_2_O]Cl · 3H_2_O or [Cu(phen)(nal)H_2_O]NO_3_ · 3H_2_O complexes.

	[Cu(hsm)(nal)H_2_O]Cl · 3H_2_O /CT-DNA		[Cu(phen)(nal)H_2_O]NO_3_ · 3H_2_O /CT-DNA	
*r*	Tm (°C)	ΔTm	Tm (°C)	ΔTm

0.0	82.7	—	82.7	—
0.096	83.5	0.8	83.4	0.7
0.28	84.9	2.2		
0.48	84.6	1.9	87.5	4.8
0.77	86	3.3	83.7	1
0.96	86.9	4.2		
